# A comprehensive analysis of oxytocin: a potential brain-based treatment to regulate obesity

**DOI:** 10.3389/fendo.2025.1708807

**Published:** 2026-01-05

**Authors:** Abdulrahman A. Alsayegh, Fauzia Ashfaq, Mohammed Bajahzer, Mohammad Y Alshaharani, Ali Saad Almutairi, Mohammad Idreesh Khan, Raghad M. Alhomaid, Mirza Masroor Ali Beg

**Affiliations:** 1 Department of Clinical Nutrition, College of Nursing and Health Sciences, Jazan University, Jazan, Saudi Arabia; 2 Department of Clinical Laboratory Sciences, College of Applied Medical Science, King Khalid University, Abha, Saudi Arabia; 3 Department of Clinical Nutrition, King Khalid General Hospital of Hafr Albatin, Hafr Albatin, Eastern Region, Saudi Arabia; 4 Department of Basic Health Sciences, College of Applied Medical Sciences, Qassim University, Buraydah, Saudi Arabia; 5 Department of Food Science and Human Nutrition, College of Agriculture and Food, Qassim University, Buraydah, Saudi Arabia; 6 Faculty of Medicine, Alatoo International University, Bishkek, Kyrgyzstan

**Keywords:** oxytocin, obesity, intranasal administration, appetite regulation, energy metabolism, food intake

## Abstract

**Background:**

Globally, obesity is a serious health issue, and oxytocin may help regulate appetite and reduce food intake, particularly in obese individuals. One promising treatment option for controlling appetite and lowering food intake is oxytocin among the people living with obesity or overweight. Based on the relevant clinical studies, this systematic review article explored the role of oxytocin and its connection with obesity.

**Methods:**

This review adhered to the Preferred Reporting Items for Systematic Reviews and Meta-Analyses (PRISMA) 2020 guidelines to ensure that our reporting was accurate and comprehensive. We examined 14 interventional studies (2015–2025) from PubMed, MEDLINE, and Scopus that had open full-text access using the keywords “Oxytocin”, “Obesity”, and “Oxytocin in Obesity”.

**Results:**

Oxytocin could be the treatment option for obesity and poor eating patterns, and it has been demonstrated that intranasal oxytocin administration reduces appetite and increases feelings of fullness, particularly in people with obesity. Administering a nasal spray of oxytocin (24 IU) can reduce appetite. Because it makes them feel fuller and less hungry, this is especially true for people with obesity. Additionally, oxytocin alters the way the brain functions in regions that govern reward and decision-making, which lessens food cravings, according to functional magnetic resonance imaging (fMRI). By influencing gut bacteria, it may also increase metabolism and assist people in controlling their eating habits. Oxytocin administration is associated with a significant reduction in weight and improved body composition. Additionally, it appears to have the potential to alleviate pregnancy related obesity. Although the initial findings are encouraging, more extensive research is necessary to confirm its effectiveness. Healthy eating and metabolism are associated with natural oxytocin levels, which suggests that it may be used to treat obesity.

**Conclusion:**

Oxytocin modulates appetite and brain reward pathways, offering a novel, brain-targeted approach to obesity treatment. Furthermore, clinical studies should explore long-term effects and optimal dosing to manage obesity.

## Introduction

Obesity has become a major threat to both developed and developing nations, causing significant health problems for men and women. Although growing evidence indicates that the neuropeptide oxytocin plays a role in controlling eating behavior and hunger, its function in the cognitive regulation of food cravings in humans remains unclear (1).

Over the past 35 years, the global prevalence of obesity has more than doubled (2). Consequently, obesity-related health issues such as type 2 diabetes mellitus, stroke, cardiovascular disease, dyslipidemia, and an increased risk of cancer have become more common (3). The hypothalamic hormone oxytocin, which is secreted into the bloodstream via the posterior pituitary, helps regulate bodily processes like eating patterns and metabolism. In rodents and nonhuman primates, oxytocin treatment reduces food intake, promotes sustained weight loss, increases energy expenditure and lipolysis, and improves glucose homeostasis (4). Several studies indicate that oxytocin effectively regulates caloric consumption and metabolic processes (5, 6). Supporting this, oxytocin receptor knockout mice develop late-onset obesity, weight gain, and poor glucose homeostasis (7).

Furthermore, oxytocin has demonstrated anti-inflammatory, wound-healing, antioxidant, and glucose uptake-enhancing properties in cardiac and stem cells (8). Both male and female oxytocin knockout mice (9) and mature male oxytocin receptor-deficient mice (10) exhibit a mildly obese phenotype. In human studies, a single intranasal dose of oxytocin (24 IU) in men increased fat oxidation (11). Zhang H et al. (2013) found that eight weeks of continuous intranasal oxytocin treatment (24 IU administered before meals and bedtime) improved lipid profiles, reduced weight, and decreased waist circumference in overweight and obese men and women (12). An increase in oxytocin signals the body to decrease calorie consumption, and alterations in oxytocin signaling can lead to weight gain (13). Oxytocin has also been shown to reduce body fat and weight by promoting lipolysis and lipid oxidation (14).

This systematic review critically assesses the evidence regarding the role of oxytocin in the pathophysiology of obesity and its emerging therapeutic potential in weight management strategies. Oxytocin may offer a novel therapeutic pathway for obesity, particularly in populations where traditional weight management is challenging. For instance, in the elderly, where obesity exacerbates sarcopenia and frailty, oxytocin’s potential to modulate metabolism could provide a dual benefit (15). This is similarly promising for women with PCOS, as oxytocin’s influence on appetite and energy balance might address the hormonal imbalances that make weight loss difficult (16, 17). Furthermore, by promoting weight loss, oxytocin therapy could potentially reduce the surgical risks associated with obesity, lessening the need for highly specialized anesthetic and surgical techniques (18). Despite advancements in personalized medicine and novel therapeutic targets like gut microbiota and neurostimulation (19–22), effective obesity treatment remains challenging due to persistent barriers including limited healthcare access and financial constraints (23).

**Figure 1:**
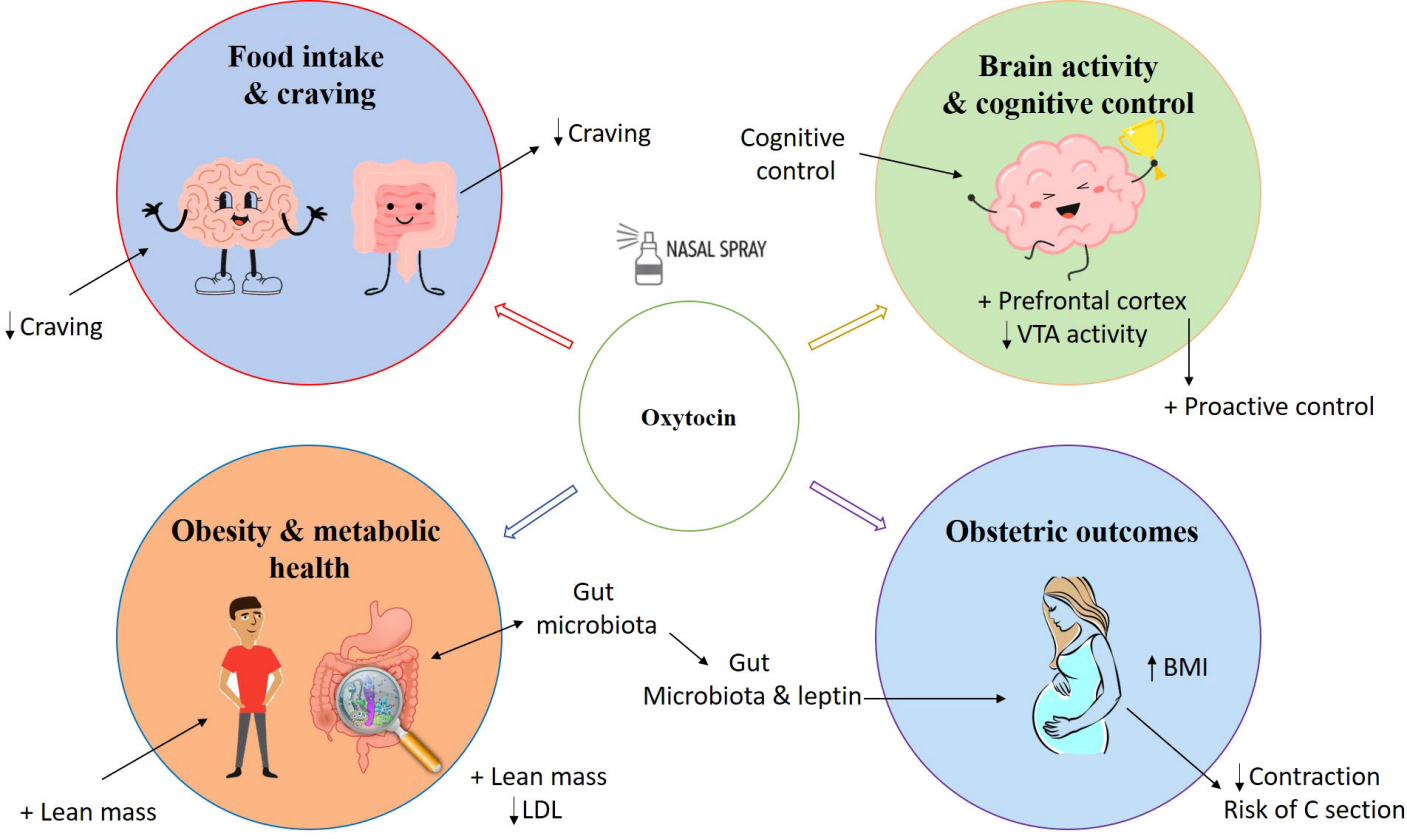
Hypothesized mechanisms through which oxytocin controls caloric intake and obesity.

This review aims to elucidate the role of oxytocin in regulating calorie intake, fat utilization, blood sugar levels, and fat burning, drawing on human research. It will focus on studying oxytocin’s effects on appetite, energy expenditure, and body weight, with particular emphasis on its potential link to obesity (**Figure 1**).

## Materials and methods

This comprehensive systematic review incorporated data from 14 clinical studies investigating the effects of oxytocin treatment in individuals with obesity. The included studies encompassed a range of designs, including randomized controlled trials (RCTs), various phases of clinical trials (I-IV), case reports, and observational studies. Furthermore, observational and cohort studies were analyzed to examine the relationships between obesity, body mass index, oxytocin treatment, food craving, and management of obesity (see **Supplementary File 1**).

### PRISMA search strategy & filters applied

**Figure 2:**
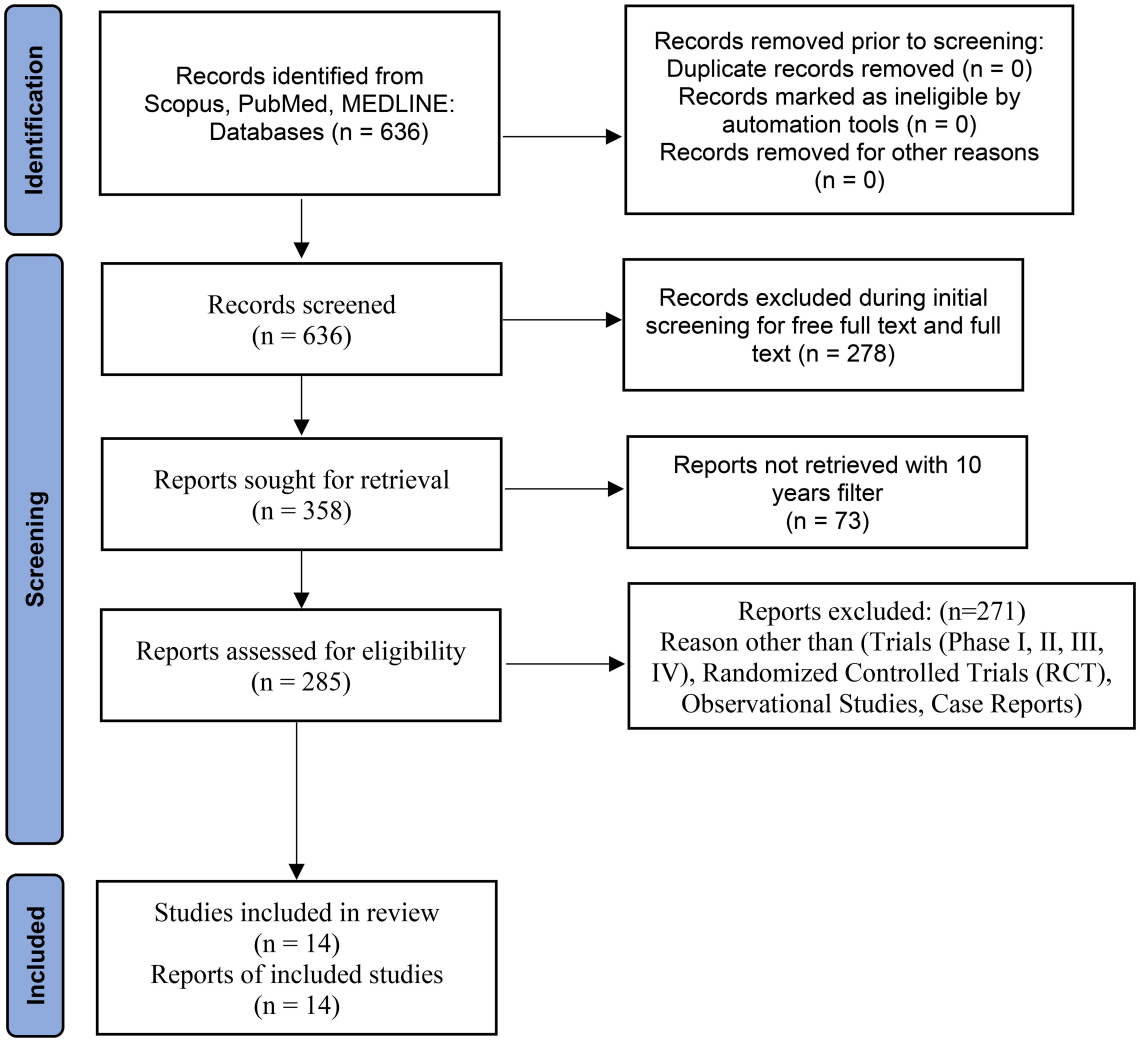
PRISMA flow diagram for study search and selection of relevant studies for the current study design.

This systematic review was conducted in accordance with the Preferred Reporting Items for Systematic Reviews and Meta-Analyses (PRISMA) 2020 guidelines. A literature search was performed using the databases PubMed, MEDLINE, and Scopus for the period 2015-2024. The search strategy utilized a combination of Medical Subject Headings (MeSH) and keywords, including “Oxytocin,” “Obesity,” “Overweight,” “Body Mass Index,” and “Adiposity,” combined with Boolean operators. The review was limited to open-access, full-text articles. Eligible study designs comprised randomized controlled trials (RCTs), clinical trials (Phases I-IV), observational studies, and case reports involving human participants (**Figure 2**). The quality and risk of bias of the included studies were thoroughly evaluated using established instruments, such as the Newcastle-Ottawa Scale for observational studies and the Cochrane Risk of Bias tool (RoB 2) for randomized trials, in order to guarantee the robustness of the synthesis.

### Study populations

The reviewed studies primarily enrolled adults with obesity (BMI ≥30 kg/m²) or overweight (BMI 25–29.9 kg/m²), with sample sizes ranging from small cohorts of 20–60 participants to larger studies of over 1,000 individuals. Most trials investigated the effects of intranasal oxytocin on metabolic, behavioral, and psychological outcomes. Smaller mechanistic studies, often employing crossover designs, assessed oxytocin’s direct impact on eating behavior, brain activity, and cognitive function. Larger observational studies focused on specific populations, such as pregnant women or primiparous women, examining correlations between oxytocin levels and maternal BMI. The research encompassed diverse demographic groups, men with diabetes, and women with dietary concerns. Collectively, these studies explored a wide spectrum of obesity-related conditions, from brain function and reproductive health to musculoskeletal and metabolic disorders, providing a comprehensive overview of oxytocin’s role in various contexts.

### Inclusion criteria

Eligible participants were generally adults (≥18 years) with overweight or obesity (BMI ≥25 kg/m²). This review included clinical trials (Phases I-IV), randomized controlled trials (RCTs), observational studies, and case reports on human. The enrolled populations were diverse, targeting specific groups such as healthy women, men with obesity, older adults with sarcopenic obesity, and individuals diagnosed with body dysmorphic disorder in social cognition studies.

### Exclusion criteria

Common exclusion criteria across studies comprised significant comorbidities (e.g., hepatic, renal, or cardiac disease), use of psychotropic or opioid medications (except in studies specifically recruiting opioid users), and pregnancy (unless the study focus was perinatal). Additional, studies were excluded if they did not meet the predefined eligibility criteria. The primary reason for exclusion was an ineligible study design, as the review was restricted to clinical trials, randomized controlled trials, observational studies, and case reports. Additionally, studies involving populations with a history of neurological or psychiatric disorders unrelated to the investigation’s primary aim were excluded to minimize potential confounding.

## Results

Intranasal oxytocin emerges as a multi-system therapeutic candidate for obesity, with evidence converging on its dual role in central appetite regulation and peripheral metabolic improvement. A single 24 IU dosage consistently lowers calorie intake by modifying important brain pathways, supporting the therapeutic potential. This is demonstrated by an increase in prefrontal cortex activation, which controls cognitive control, and a decrease in ventral striatum activity, which processes reward. Interestingly, compared to those of normal weight, this impact is more noticeable in people with obesity, as evidenced by a 15% higher reduction in calorie consumption. Additionally, an 8-week program has been demonstrated to sustain a considerable reduction in calorie intake while no change in body weight as compared to a placebo. Important metabolic metrics, such as total fat mass, abdominal visceral fat, liver fat, and resting energy expenditure, did not significantly improve as a result. A single test meal showed a little decrease in calorie intake, but this did not result in weight loss, suggesting the intervention was mainly unsuccessful. Oxytocin also enhances proactive cognitive control, which is the capacity to anticipate and resist food signals. Oxytocin has direct metabolic benefits in addition to its central effects. Daily treatment improved lipid profiles, lowering LDL and increasing lean mass in older persons with sarcopenic obesity, according to a 12-week experiment. However, it has also been observed that the level of endogenous oxytocin impacted the metabolic health and is consistent with observational research that links greater endogenous oxytocin levels to better metabolic parameters and healthier gut microbiota.

Obesity is associated with a functional deficit in the oxytocin system, which emphasizes the therapeutic significance of oxytocin signaling in peripartum care. There is a direct correlation between weaker uterine contractions, a higher risk of protracted labor and cesarean birth, and lower and less sustained oxytocin levels during labor in women with higher BMI. This reveals a viable physiological basis for recognized obesity-related obstetric problems.

The relationships between oxytocin levels, the makeup of the gut bacteria, and metabolic hormones like leptin suggest that oxytocin plays a part in the gut-brain axis. A wider neuromodulatory role that may be pertinent to treating emotional eating patterns is also suggested by its extra ability to enhance social and emotional cognition.

All of the evidence points to intranasal oxytocin as a special agent that combats obesity by improving body composition and metabolism, reducing appetite by rebalancing brain reward and control pathways, and possibly altering gut-brain and emotional pathways (**Table 1**).

**Table 1:** An outline of the main studies evaluating the role of oxytocin in obesity, metabolic control, and related effects.

First author (year)	Population/design	Oxytocin dose/route	Main outcome
Striepens N (2016) (1)	30 healthy women, RCT with fMRI	24 IU intranasal (1 dose)	Reduced food cravings, increased prefrontal cortex activity (cognitive control), and decreased ventral striatum activity (reward).
Thienel M (2016) (24)	20 men (10 obese, 10 normal-weight), RCT, crossover	24 IU intranasal (1 dose)	Reduced food intake by ~15% in obese men, a significantly stronger effect than in normal-weight men.
Plessow F (2018) (45)	25 overweight/obese men, placebo-controlled fMRI RCT	24 IU intranasal (1 dose)	Reduced brain activity in reward areas and increased activity in cognitive control regions in response to food cues.
Barengolts E (2018) (40)	60 African American men with type 2 diabetes, observational cross-sectional	Endogenous levels (no intervention)	Circulating oxytocin levels were correlated with specific gut microbiota species and leptin.
Fang A (2019) (30)	25 adults with body dysmorphic disorder (BDD), double-blind, placebo-controlled crossover RCT	24 IU intranasal (1 dose)	Improved performance in emotional recognition and theory-of-mind tasks, indicating enhanced social cognition.
Kerem L (2020) (29)	30 men with overweight or obesity, placebo-controlled fMRI RCT	24 IU intranasal (1 dose)	Reduced functional connectivity between the VTA and food motivation regions, suggesting modulation of reward pathways.
Dalbye R (2021) (39)	1000+ nulliparous women, large cohort study	Endogenous levels (no intervention)	Higher BMI was associated with increased risk of cesarean section, prolonged labor, and neonatal complications.
Plessow F (2021) (27)	40 men with overweight or obesity, double-blind, placebo-controlled crossover RCT	24 IU intranasal (1 dose)	Increased proactive cognitive control, enhancing the ability to anticipate and inhibit food responses.
Espinoza SE (2021) (37)	30 older adults with sarcopenic obesity, Pilot RCT	24 IU intranasal (daily) for 12 weeks	Increased lean muscle mass by ~5% and reduced LDL cholesterol by ~10 mg/dL; treatment was well-tolerated.
Ramö Isgren A (2021) (46)	200 pregnant women, prospective observational	Endogenous levels (no intervention)	Higher maternal BMI was associated with lower oxytocin levels and a greater need for oxytocin augmentation during labor.
Hautakangas T (2022) (47)	200 women, blinded analysis of an RCT cohort	Endogenous levels (no intervention)	Obesity was associated with weaker uterine contractions and prolonged labor duration.
Wronski ML (2022) (26)	~120 obese adults (planned), 8-week double-blind RCT	Intranasal oxytocin (dose not stated)	Study protocol; results on safety, weight change, and metabolic markers are pending.
Ramö Isgren A (2023) (38)	200 pregnant women, prospective observational	Endogenous levels (no intervention)	Higher maternal BMI was associated with lower and shorter-lasting oxytocin levels during labor.
Plessow F (2024) (25)	61 adults with obesity, randomized double-blind placebo-controlled trial	24 IU intranasal (four times daily for 8 weeks)	Reduction in food intake, No significant difference in body weight change vs placebo; no beneficial effects on body composition or energy expenditure.
Colonnello E (2025) (28)	50 adults with obesity, observational exploratory study	Endogenous levels (no intervention)	Higher endogenous oxytocin levels were associated with healthier eating habits and improved lipid/glucose profiles.

### Central neuromodulatory effects on appetite and eating behavior

The included studies provide consistent evidence that intranasal oxytocin, typically at a 24 IU dose, modulates key neural circuits governing food motivation and cognitive control. Neuroimaging findings demonstrate a dual mechanism a reduction in reward system activity coupled with an enhancement of prefrontal regulatory regions. By centrally suppressing hedonic eating through the brain’s reward system, oxytocin helps people lose weight. The mechanism involves two actions on the mesolimbic dopamine pathway: it weakens the functional connectivity between the dopamine-producing ventral tegmental area (VTA) and the striatum (25) and directly decreases neural activity in the ventral striatum, a region crucial for food reward (1). The “wanting” of high-calorie meals is diminished by this combination inhibition of circuit activity and communication, which lowers caloric intake and causes fat mass reduction. Concurrently, it increased activation in prefrontal cognitive control areas (1, 45). This neural recalibration translated to significant behavioural outcomes. A single dose of oxytocin reduced caloric intake by approximately 15% in men with obesity, an effect significantly more potent than in normal-weight individuals (24). Furthermore, oxytocin was found to enhance proactive cognitive control the anticipatory inhibition of food responses in overweight and obese men (27). Intranasal oxytocin administration (24 IU, four times a day, over 8 weeks) didn’t prove effective for losing weight or enhancing body composition in obese adults, even though it slightly lowered how many calories they consumed in the short term, implying it might not be very useful for treating obesity (25).

### Peripheral metabolic and physiological effects

Beyond its central actions, oxytocin exerted beneficial effects on body composition and metabolic parameters. In a 12-week pilot RCT involving older adults with sarcopenic obesity, daily intranasal oxytocin (24 IU) significantly increased lean body mass by approximately 5% and reduced LDL cholesterol by an average of 10 mg/dL (37). Observational data corroborate these interventional findings, indicating that higher endogenous oxytocin levels are correlated with healthier eating patterns and more favourable lipid and glucose profiles (28). An exploratory study also identified significant correlations between circulating oxytocin levels, specific gut microbiota species, and leptin in men with type 2 diabetes, suggesting a potential role for oxytocin in gut-brain axis signaling (40).

### Oxytocin dysregulation in obstetric populations

Evidence from obstetric cohorts indicates that obesity is associated with a functional impairment of the endogenous oxytocin system, with direct clinical consequences. Large cohort studies established that a higher maternal Body Mass Index (BMI) is a significant predictor of prolonged labour duration, weaker uterine contractions, and an increased risk of cesarean delivery (39; 47). Investigating the underlying mechanism, Ramö Isgren et al. (38) demonstrated that women with a higher BMI exhibited both lower circulating plasma levels of oxytocin and a shorter duration of oxytocin pulsatility during labour, providing a physiological explanation for the labour dysfunctions observed in this population (**Table 2**).

**Table 2:** Summary of clinical studies investigating role of oxytocin in obesity and associated outcomes.

First author (year)	Study focus/purpose	Population	Study design & sample size	Intervention & dosage/duration	Primary outcomes	Key findings	Limitations/notes
Striepens (2016) (1)	Food craving, cognitive control, and brain response.	Healthy women	RCT, crossover + fMRI *N=30*	24 IU intranasal (1 dose)/Single session	Food craving, brain activation	↓ Food craving, ↑ prefrontal cortex activity (cognitive control), ↓ ventral striatum activity (reward).	Small sample, healthy women only.
Thienel (2016) (24)	Food intake inhibition in obese vs. normal-weight men.	Men (10 obese, 10 normal-weight)	RCT, crossover *N=20*	24 IU intranasal (1 dose)/Single session	Food intake	↓ Food intake by ~15% in obese men; effect significantly stronger than in normal-weight men.	Small sample, male-only.
Plessow (2018) (45)	Brain modulation in response to food cues.	Overweight/obese men	RCT, placebo-controlled + fMRI *N=25*	24 IU intranasal (1 dose)/Single session	Brain activity to food cues	↓ Reward signals (VTA, striatum), ↑ cognitive control areas (prefrontal cortex).	Small sample.
Barengolts (2018) (40)	Correlation between gut microbiota, leptin, and oxytocin.	African American men with Type 2 Diabetes	Observational cross-sectional *N=60*	Endogenous levels (no intervention)/Cross-sectional	Gut microbiota, hormone levels	Oxytocin levels correlated with specific gut microbiota species and leptin.	Correlational only, no causation.
Fang (2019) (30)	Social cognition in Body Dysmorphic Disorder (BDD).	Adults with BDD	RCT, placebo-controlled crossover *N=25*	24 IU intranasal (1 dose)/Single session	Social cognition, emotional recognition	↑ Emotional recognition and social processing.	Small sample, BDD-specific population.
Kerem (2020) (29)	Brain functional connectivity related to eating control.	Overweight/obese men	RCT, placebo-controlled + fMRI *N=30*	24 IU intranasal (1 dose)/Single session	Brain functional connectivity	↓ Functional connectivity between VTA and food motivation/reward regions.	Small sample, needs clinical correlation.
Dalbye (2021) (39)	Association between maternal BMI and obstetric/neonatal outcomes.	Nulliparous women	Large cohort study *N=1000+*	Endogenous levels (no intervention)/Pregnancy	Labor outcomes, cesarean risk	↑ BMI = ↑ Risk of cesarean section, prolonged labor, and neonatal complications.	Observational.
Espinoza (2021) (37)	Effect on body composition (muscle mass) and lipids.	Older adults with sarcopenic obesity	Pilot RCT *N=30*	24 IU intranasal daily/12 weeks	Lean mass, LDL cholesterol	↑ Lean mass (~5%), ↓ LDL cholesterol (~10 mg/dL); treatment was well-tolerated.	Small pilot study.
Plessow (2021) (27)	Proactive cognitive control over eating.	Overweight/obese men	RCT, double-blind, placebo-controlled crossover *N=40*	24 IU intranasal (1 dose)/Single session	Proactive cognitive control	Improved proactive control (anticipatory inhibition of food responses).	Small sample.
Hautakangas (2022) (47)	Obesity’s effect on uterine contractile activity during labor.	Women	Blinded RCT cohort analysis *N=200*	Endogenous levels (no intervention)/Labor	Uterine contractile activity	Obesity associated with weaker uterine contractions and longer labor duration.	Secondary analysis.
Wronski (2022) (26)	Trial to assess safety and efficacy of intranasal oxytocin.	Adults with obesity (planned)	RCT, double-blind, placebo-controlled (protocol) *Planned N=~120*	Intranasal oxytocin (dose not stated)/8 weeks	Safety, weight change	Study protocol; rationale and methods published. Results pending.	Results pending.
Ramö Isgren (2023) (38)	Impact of maternal BMI on endogenous oxytocin levels during labor.	Pregnant women	Prospective observational *N=200*	Endogenous levels (no intervention)/Labor	Plasma oxytocin levels	↑ BMI = ↓ Oxytocin levels and shorter duration of oxytocin pulses during labor.	Observational only.
Plessow (2024) (25)	Effect on energy intake, weight, and eating behavior.	Adults with obesity	RCT, double-blind, placebo-controlled crossover *N=~100*	24 IU intranasal/8 weeks	Energy intake, body weight	↓ Energy intake (~10%), ↑ satiety, but no significant weight loss vs. placebo.	Contrasts with short-term intake studies; needs longer-term data.
Colonnello (2025) (28)	Correlation between endogenous oxytocin, diet, and metabolic health.	Adults with obesity	Observational exploratory study *N=50*	Endogenous levels (no intervention)/Cross-sectional	Oxytocin levels, metabolic markers	↑ Endogenous oxytocin linked to healthier diet, better lipid and glucose profiles.	No causal inference.

## Discussion

### Evidence for appetite suppression and weight loss

Comprehensive research has made the role of oxytocin in lowering appetite and managing body weight. The trial by Thienel and colleagues (2016) demonstrated the potency of oxytocin’s appetite-suppressive effects. Participants were randomly allocated to groups without knowing if they got a placebo or the genuine medication. Each subject received all treatments in a random order as part of the research design. The findings demonstrated that men who were obese reduced their food intake significantly more than men who were of normal weight, underscoring the potential significance of oxytocin in addressing unhealthy eating practices (24) suggested that the acute appetite suppression has been successfully translated into long term weight management outcomes. It has been revealed that the 8-week randomized controlled trial demonstrated that daily intranasal oxytocin considerably reduced caloric intake, this did not yield significant weight loss or improve metabolic health markers such as body fat composition, liver fat, or energy expenditure (25). Wronski et al. (26), who developed the framework for assessing oxytocin’s effectiveness in treating obesity, previously described the thorough research design and methods for such long-term investigations (26). He said that research conducted over a short period suggests that oxytocin decreases the amount of food consumed, the longer duration effectiveness is still uncertain. Research for eight weeks to find out whether the immediate positive effects can lead to lasting weight reduction and better metabolic function in everyday situations. The detailed structure of this study will offer crucial insights into whether oxytocin can be a practical treatment for obesity (26). Oxytocin improves cognitive elements of eating behavior in addition to its direct impact on calorie intake. Oxytocin’s effect on cognitive control was explicitly examined by Plessow et al. (27), who found that acute treatment greatly improved proactive control in males who were overweight or obese a critical cognitive function for predicting and averting dietary failures (27). This behavioral improvement may stem from fundamental physiological relationships, as Colonnello et al. (28) identified positive correlations between circulating oxytocin levels and healthier eating behavior patterns in individuals with obesity, suggesting endogenous oxytocin may naturally support better dietary self-regulation (28). The convergence of evidence from acute laboratory studies, long-term clinical trials, and observational research strongly positions oxytocin as a multifaceted agent for appetite and weight management. Oxytocin demonstrates inconsistent appetite-suppressing effects across populations, with initial benefits often diminishing over time. Long-term clinical trials show no significant weight loss difference compared to placebo, while contextual factors further limit its reliability as an obesity treatment (4, 6).

### Neurobiological mechanisms

The impact of oxytocin on behavior arises from its complex influence on the brain’s communication systems within areas that manage pleasure, feelings, and decision-making. Brain scans have repeatedly shown that oxytocin can correct abnormal brain activity related to food triggers in individuals with obesity. Kerem et al. (29) provided compelling evidence that oxytocin reduces functional connectivity between reward-related brain regions during fasting states in men with overweight and obesity, effectively decoupling the neural circuitry that drives compulsive eating behavior (29). Plessow et al. (28) employed fMRI to demonstrate oxytocin’s dual mechanism of action: simultaneously decreasing activation in subcortical food motivation pathways while enhancing engagement of prefrontal cognitive control regions during food cue exposure (28). This bidirectional modulation represents a unique neurobiological profile, diminishing bottom-up hedonic drives while strengthening top down regulatory control. Oxytocin’s capacity to improve social and emotional thinking skills, including understanding others’ perspectives and recognizing emotions, tackles the unhelpful thought patterns and long-term stress that might contribute to eating disorders (30, 31). Through influencing these systems, oxytocin then lessens the overactivity of the HPA axis, which is a crucial process closely associated with eating for pleasure and the accumulation of abdominal fat in obesity (30, 31). These parallel effects on social and food-related cognition suggest oxytocin may act through shared neural mechanisms to improve behavioral regulation across multiple domains, positioning it as a broad-spectrum neuromodulator with particular relevance for disorders characterized by compulsive behaviors. The clinical relevance of oxytocin for obesity is uncertain. Methodological issues like small sample sizes and a lack of replication undermine the reliability of findings, while observed changes in brain activity may be transient pharmacological effects (32, 33). Oxytocin effect is highly context-dependent, varying significantly by individual and situation rather than providing reliable modulation (34). Consequently, its impact on social-emotional domains does not guarantee efficacy for eating behavior modification, as demonstrated in reviews of its effects on mood disorders (36). Administration of oxytocin downregulates food intake as well as body mass in both animals and humans. By combining its social and metabolic influences, oxytocin can be considered as a potential drug for the treatment of abnormal eating (35). Chen et al. (2021) suggested that intranasal oxytocin significantly reduced food intake in nonpsychiatric subjects. While a single dose of intranasal oxytocin was generally safe, and suggested that determining its long-term effects on body weight and mental status are necessary before it can be recommended for routine obesity management (36).

### Metabolic effects beyond weight

Oxytocin may be used to treat a wide range of metabolic, body composition, and other physiological problems, not just helping people lose weight. The groundbreaking randomized controlled trial by Espinoza et al. (37) on older people who had sarcopenic obesity showed that intranasal oxytocin administration not only helped them gain more lean muscle, but it also greatly lowered their LDL cholesterol levels, which are two serious health issues that often happen at the same time in older people and those with metabolic diseases (37). It has been observed that the oxytocin signaling affects reproduction, demonstrating a significant association between maternal BMI and dynamic blood oxytocin levels during childbirth. This implies that obesity can disrupt normal oxytocin patterns in key physiological processes (38). Findings align with the epidemiological work of Dalbye et al. (39), whose large cohort study established connections between elevated BMI and adverse obstetric outcomes, potentially mediated by altered oxytocin function (39). The gut-brain axis represents a promising frontier for understanding oxytocin’s systemic effects and has been identified significant relationships between gut microbiota composition, circulating leptin, and oxytocin levels in African American men with diabetes, suggesting that complex endocrine-microbiome interactions may influence metabolic health (40). This concept is further elaborated by Aydin et al. (41), who proposed a mechanistic framework through which oxytocin might modulate gut microbiota to influence energy homeostasis and metabolic regulation (41). The most dramatic demonstration of oxytocin’s clinical potential comes from Hsu et al. (42), who successfully employed combination therapy with oxytocin and naltrexone to treat hypothalamic obesity following craniopharyngioma resection, achieving significant weight reduction in a condition notoriously resistant to conventional interventions (42). This case underscores oxytocin’s potential as a targeted therapy for even the most challenging forms of obesity, acting through multiple synergistic pathways to restore metabolic balance. Oxytocin administration shows only modest reductions in calorie intake, with its metabolic benefits appearing largely dependent on concomitant weight loss (43). Furthermore, the long-term cardiovascular safety of chronic oxytocin use remains inadequately studied (44). Preclinical studies in rodent models of obesity have indeed explored a wider range of doses and administration routes, revealing a more nuanced picture. While chronic subcutaneous infusion or repeated intraperitoneal injections of oxytocin consistently demonstrate dose-dependent reductions in food intake, body weight, and improvements in glucose homeostasis and lipid metabolism (5, 6). A study by Lawson et al. (11) found that a lower dose of 18 IU was sufficient to reduce caloric intake in men, suggesting that the effective dose for acute appetite suppression may be at or below the commonly used 24 IU (11). Plessow et al. (25), which used a more intensive regimen of 24 IU administered four times daily. While this regimen led to a reduction in energy intake, it did not translate to significant weight loss or improved body composition (25). Overcoming the short half-life of native oxytocin, research is exploring long-acting oxytocin receptor agonists. Preclinical studies with such compounds show promising, potent, and sustained reductions in body weight and food intake, outperforming native oxytocin (5). Although its bioavailability and the relationship between peripheral dosage and central action are still poorly understood, the intranasal route is thought to provide direct delivery to the central nervous system via olfactory and trigeminal pathways. According to preclinical research, sustained metabolic advantages similar to those shown with chronic peripheral injection in animals may need greater or more frequent doses than the normal 24 IU.

## Conclusion

Oxytocin, a neurohormone produced in the hypothalamus, demonstrates significant potential in obesity management by modulating central feeding pathways. Intranasal administration reduces appetite, enhances satiety, and decreases cravings for high-calorie foods, with effects being more pronounced in individuals with obesity. Beyond appetite regulation, oxytocin may offer metabolic benefits, including the preservation of muscle mass and improvements in lipid and glucose profiles, particularly in conditions like sarcopenic obesity. While these findings position oxytocin as a promising therapeutic adjunct, further research is essential to establish standardized dosing and confirm long-term efficacy and safety.

### Limitations and future directions

Small-scale, short-term studies with homogeneous cohorts reduce generalizability and obscure long-term safety and efficacy, which limits the available data. The pharmacokinetic limits of natural oxytocin, especially its short half-life, are highlighted by the translational gap between strong preclinical findings and moderate clinical outcomes. Large-scale, long-term studies must be given top priority in future research in order to demonstrate clinical validity and clarify causative pathways. Systematic dose-finding studies, the creation of long-acting oxytocin receptor agonists with improved central delivery, the discovery of predictive biomarkers for tailored treatment, and research into oxytocin’s function in gut-brain axis regulation are crucial future steps.

## Data Availability

The data analyzed in this study is subject to the following licenses/restrictions: NA. Requests to access these datasets should be directed to mirzamasroor.alibeg@alatoo.edu.kg.
